# “Understanding growth convergence in India (1981–2010): Looking beyond the usual suspects”

**DOI:** 10.1371/journal.pone.0233549

**Published:** 2020-06-02

**Authors:** Aparna P. Lolayekar, Pranab Mukhopadhyay

**Affiliations:** 1 Department of Women’s Studies, Goa University, Taleigao Plateau, Goa; 2 Goa Business School, Goa University, Taleigao Plateau, Goa; Institute of Economic Growth, INDIA

## Abstract

The literature on growth convergence has focused to a great extent on the role of initial incomes as a primary determinant of long-term growth outcomes. Expanded versions of growth models have used other explanators to unpack the growth process. In this paper we add to the literature in two significant ways: (a) we use socioeconomic variables that are sometimes overlooked in explaining growth (such as, political stability and political alliance, social heterogeneity, and demographic distribution), and (b) we demonstrate that earlier analyses may be overlooking the problem of normality and endogeneity in regression models (and we provide alternate methods like instrumental variable and distribution dynamics to control for these). In this paper we analyze the per capita income growth at the subnational level in India for the period 1981–82 to 2010–11 using an expanded growth framework. We find that initial incomes, the ratio of working age group to total population, political stability and alliance, and the extent of development expenditure play a positive and significant role in predicting growth. We also find that, contrary to popular belief, the presence of marginalized groups—namely Scheduled Castes and Scheduled Tribes—have not been a hindrance to growth of per capita incomes in states. Our findings on the influence of social institutions may have significant implications for a public policy of affirmative action in India. The results on the impact of development expenditure on growth is also important for states seeking to increase their growth rates through policy intervention.

## Introduction

One of the primary objectives of public policy in India is to reduce poverty and inequality by promoting economic growth as a vehicle [1]. In India, not all regions and states have grown at the same pace nor has the decline in poverty rates been uniform over the last few decades [2,3]. There is a consensus that economic growth alone cannot bring down poverty [4,5]. The problem of deprivation is intertwined with inherited economic hierarchies, religious and linguistic disparities, and political networks [2,6,7]. These may act as barriers to transfer of benefits to the poor [8]. Some studies indicate that both social factors (different castes like Schedule Caste [SC], Schedule Tribe [ST] and Other Backward Caste [OBC]) and political factors (such as alliances between state and central political parties in a federal system) could influence per capita incomes [9–11]. Historically established social hierarchies like the caste system are seen as key drivers of economic inequality [12–14]. In recognition of their historically disadvantaged status, the Constitution has provided for positive discrimination for such groups.

The 2011 Census of India found that 16.6 percent of the population belongs to the SC, and 8.6 percent belongs to the ST, community. There is wide heterogeneity in these proportions at the state level. In absolute terms, the state which has the highest SC population is Uttar Pradesh (41.4 million, which is 20 percent of its population). The second largest population in absolute numbers is Madhya Pradesh (11.3 million, which is about 15 percent of its population). In percentage, Punjab has the highest at about 32 percent even though the absolute population of SCs (8.9 million) is lower than other states. Mizoram, at the other end of the spectrum, has only 0.1 percent of its population from the SC community totaling about 1,218 in absolute numbers. Arunachal Pradesh, Nagaland, Lakshadweep, and Andaman and Nicobar Islands have not reported any significant SC population.

The Census also records that Madhya Pradesh has the highest number of STs (15.3 million, which is about 21 percent of its population). The northeastern states, even though smaller in population size, have a very high percentage of ST population, led by Mizoram (95 percent), Meghalaya and Nagaland (86 percent), Manipur (43 percent), Sikkim (34 percent), and Tripura (32 percent). Assam, which is also considered contiguous as part of this geographical space, reported only 13 percent of its population as STs. Among the Union Territories, Lakshadweep has the highest percentage of STs (95 percent). Punjab, Haryana, Delhi, Chandigarh, and Puducherry have not reported any significant ST populations.

This heterogeneity in demographic distribution has significant implications. A large number of policies for the upliftment of historically deprived groups have been devised both at the central and state levels. The northeastern part not only has a large ST population but is also geographically difficult to access. There are also issues of cross-border conflict in these areas that add to the challenges for development planning in this region. However, we will not discuss these specific policies here as it would take us away from the focus of this paper.

The existence of caste-based voting, political decision-making, distribution of privileges, and public expenditure based on caste identities is a common phenomenon in India [15,16]. This, therefore, raises the question: What has been the growth outcome of the states which have large proportions of historically disadvantaged (hereafter, marginalized) groups?

In growth economics, the key economic issue is whether the rich countries or regions will remain rich and the poor remain poor for various decades or whether the initially laggard ones will ever grow faster and catch up with the rich ones in per capita terms. The empirical debate on economic disparities (convergence) has conventionally centered around the inverse relationship between the growth of per capita income and the initial levels of income across countries [13,17] or across the regions within countries [13,14]. The original idea dates back to the notion of β-convergence. The belief was that if regions have unequal incomes to start with, they will experience unequal growth rates in the short run but will converge to a steady-state solution [18]. The flexibility in capital-output ratio would help avoid the knife-edge problem predicted by the Harrod-Domar growth models [19]. Solow’s model [18] also moved the focus away from the savings rate as the determinant of the growth rate to technological change. The savings rate was only expected to have a level-effect on per capita levels of the Gross Domestic Product (GDP). This established a basic framework to explain a negative relation between the initial per capita incomes and the growth rate. Given technology, it is the diminishing marginal returns to capital that would move economies to reach unique steady, stable equilibrium. It follows, therefore, that relatively poor countries or regions with low capital-labor ratios will experience high marginal returns on capital. They would attract greater investment and thereby grow faster. The initial β-convergence hypothesis relied on a strong notion of convergence called the absolute or the unconditional convergence. This required that parameters such as the saving rate, technological progress, depreciation, and the rate of growth of population are same across the regions and countries. However, all these parameters may not be the same across the countries. This would imply that every country would not converge to a common steady-state level but may converge to different steady-state levels determined by the parameters pertaining to those countries. This hypothesis was termed as conditional convergence [20].

The proposition of unconditional convergence has been tested by many researchers, and there are far too many to exhaustively discuss here. We gather that some have found convergence of countries towards the steady-state levels [13,21], while many others have argued that the economies have diverged from the steady-state levels [14,22–24].

The empirical debate on economic disparities (convergence) was revived when an inverse relationship was found between the growth of per capita income and the initial levels of income across countries using a small sample of countries [17]. Some of the significant contributions that followed looked both at inter-country as well as intra-country convergence. For example, one study examined a set of 98 countries during the period 1960–85 [13]. The relationship between the average growth rate of per capita real GDP and the initial value of real per capita (in 1960) was not significant. While there was no evidence of absolute convergence across these countries, there was supportive evidence for conditional convergence.

Long term intra-country evidence from the United States of America (48 contiguous US’ states) showed absolute convergence [14]. An expanded study examined a sample of 110 countries, the subsample of the Organisation for Economic Cooperation and Development (OECD) countries, the states within the US, the prefectures of Japan, and regions within several European countries, for the period 1960–1990 [25]. The study found that the cross-country distribution of income did not shrink during this period. The poorer countries did not grow faster than the rich ones. However, regions within the US, Germany, Japan, the United Kingdom, and France showed evidence of absolute, conditional as well as “σ” convergence. Similar results were reported for pooled and cross-section but significantly different results for the panel estimation for the non-oil country sample of 96 countries, INTER-75 countries, and OECD 22 countries [22].

The reason many of these early studies focused on developed countries is because of data availability issues. Unlike developed countries, the developing countries did not exhibit convergence. In China, the rise in inequality matched the different political–economic periods in Chinese history [23]. The heavy-industry development strategy in China led to a rural–urban gap in the pre-reform period, while openness and decentralization led to the rapid increase in inland–coastal disparity in the reform period of the 1980s and ‘90s.

Brazilian municipalities exhibited two convergence clubs, with municipalities of the north and northeast regions forming a low-income club, while the municipalities of the center-west, southeast, and south regions in Brazil forming the high-income club over the period 1970–1996 [24]. If the impact of trade openness is considered, then the growing inequalities in Mexico during the period 1980–2000 are associated with trade reforms [26].

In India, as we have discussed earlier, there are vast differences among the states with regard to demography, geographical factors, pattern of urbanization, stock of human capital, state institutions, and public expenditure polices, among others. Therefore, it is quite possible that the steady states might differ across the regions.

Concerns about explaining growth outcomes with just one explanatory factor (namely, initial income) encouraged researchers to use additional covariates. The expanded growth equations use initial income as one of the main explanatory variables along with population growth [27], educational attainment [13] and human capital [28], infrastructure [29], public and private investment [28], public expenditures [30], and center-state government transfers [31,32].

The New Growth Theory (NGT) recognizes that the government’s budget policy can influence the long-term growth rate through its decisions on priority-based public spending in social sectors [33]. In the Indian context, evidence exists that policy intervention could condition growth [34]. Development expenditures would raise human capital as well as the quality of life of the people. Human capital could result in increasing returns to scale as postulated in the NGT. It is common to treat government expenditure as an exogenous variable in growth regressions as it is assumed to be determined by discretionary factors [13,31,35,36]. Some authors have explicitly accounted for it in the expanded growth model and found that it influences regional growth [34,37]. In this paper, we consider a sample of these variables to understand the Indian growth process over a three-decade period from 1981–82 to 2010–11.

We add to the empirical growth literature in India in two significant ways: (a) we consider the effect of socioeconomic variables (such as, political stability and political alliance, social heterogeneity, and demographic distribution); and (b) we overcome the problem of normality and endogeneity in growth regression models by using an instrumental variable approach (for presence of endogeneity) and distribution dynamics (for violation of normal distribution assumption). Our results indicate that socioeconomic variables such as the ratio of working age group to total population, political stability and alliance, and the extent of development expenditure have a positive and significant impact on growth. The presence of large marginalized groups—namely SCs and STs—does not hinder growth in states that have a larger share of these groups.

The rest of the paper is organized as follows. First, we discuss the theoretical framework of convergence, and propose an ‘instrumental variable’ (IV) approach and the ‘distribution dynamics’ approach as empirical strategies to test for convergence. We also discuss the data used for the empirical analysis in this section. We then present our results followed by our analysis. Our paper concludes with a discussion of our findings.

## Material and methods

The existing empirical literature on cross-country growth rates has been questioned for using inconsistent estimation procedures [38–40]. There are two well-known problems with the regression-based empirical growth analysis—one is the independence of explanatory variables (endogeneity), and the second is the normality of distribution of the error term. Initial income could be endogenously determined, and so the estimated coefficient of initial income would be biased. If the normality assumption is violated, the reliability of confidence intervals and significance tests for coefficients could be questioned.

In order to overcome the problems caused by presence of endogeneity, we propose an IV approach in an expanded growth equation [38] using additional variables such as social stratification, political alliance, political stability and development expenditures as instruments. Caste is a historically determined sociodemographic category [15] and not determined by economic processes. It is, therefore, a noncontentious instrument. Political alliances between the center and the state government are determined by electoral processes where millions of heterogeneous voters elect the governments at the center and the states independently. Similarly, political stability in the states would depend on the mandate of the voters through the electoral process as discussed above. These political variables, therefore, are potentially good instruments and unlikely to be correlated to growth across subnational units (states) over time. We have confirmed that the correlation between growth, political alliance, and political stability is insignificant in our study. Some studies have used development expenditure in regression analysis to predict growth among states in India [34,37]. Our data suggests that lagged per capita development expenditure by states is not correlated to growth but is correlated to initial per capita income. These variables, therefore, are potentially good instruments in the IV model (S1 Text). The expanded growth equation allows us to have a more nuanced analysis of how initial per capita incomes could be influenced by social stratification (caste system), political alliances, political stability, and development expenditures.

Regression analysis assumes that when multiple variables are used as covariates the error term is normally distributed [41]. However, this assumption is not fulfilled sometimes. We tested for normality of errors in the basic and expanded growth equation and found that the normality assumption is not fulfilled (S2 Text and S1 Table). In order to overcome this problem of the normality assumption, we model the evolution of relative income distribution for Indian states within the framework of social stratification using the distribution dynamics methodology [10,12]. As discussed earlier, the SCs and STs are historically-disadvantaged groups as recognized in the Constitution of India. They continue to suffer from extreme social, educational, and economic backwardness arising out of isolation and the age-old practice of untouchability [42,43]. The list of SCs and STs is notified for each state and union territory (UT) [42,44].

### Regression-based approach

The basic idea of convergence in growth rates among countries is that if regions are similar in structural parameters such as preferences and technology, then poor regions will tend to grow faster than rich ones [17]. This occurs because the marginal product of capital is low in high-income regions as they have a high capital-labor ratio and vice versa for low-income regions [20]. Numerous empirical techniques have evolved to test the convergence theory and the most widely used model takes the following form [13]:

$$Y_{i,t}=\beta_{0}+\beta_{1}x_{it-1}+u_{i,t}$$ (1)

where, *Y*_*i*,*t*_ is the *i*^*th*^ region’s annual average growth rate of per capita income between period “*t-1*” and *“t*”, “*x*_*it*−1_” is the *i*^*th*^ region’s per capita income at the initial time *t-1* and *u*_*i*,*t*_ is the random error term. A negative value of “β_1_” indicates convergence, while a positive value implies divergence. In case *β*_1_ = 0 then the initial income does not affect growth.

When all the regions grow at the same rate, we have absolute convergence. This is expected to occur when all regions have the same technology, population growth rate and savings propensity, capital-labor ratio leading to a common output per capita, consumption per capita, and growth rate [45]. Since regions differ in many of these conditions, there could be different steady states for different regions—a situation described as conditional convergence [21]. The other popular method to test for convergence is based on the dispersion of income and is called “σ-convergence”. However, in this paper, we do not elaborate on this.

Many studies have tested for convergence among the Indian states. Some have found evidence of convergence [30,46], while a majority have reported divergence over the last few decades [27,29,31]. Conventional growth estimates may be inconsistent as the underlying empirical model could suffer from issues of endogeneity. The simultaneity bias (when changes in the dependent and the explanatory variables are jointly determined) cause the explanatory variables and the stochastic disturbance terms to be dependent [47].

One mechanism to overcome this problem is to use the IV approach. A valid instrument (Z_*i*_) would isolate the part of the endogenous variable that is not related with the error term (S1 Text). This unrelated part (to the error term) is used to estimate the impact on the dependent variable. To operationalize this technique, the Two-Stage Least Square (TSLS) method is used (S3 Text). In the first stage we regress *x*_*i*,*t*−1_ on *Z*_*i*,*t*−1_ to get the predicted value of ${\hat{x}}_{i,t-1}$ (see Eq 2).

$${\hat{x}}_{i,t-1}=\alpha_{0}+\alpha_{1}Z_{i,t-1}+u_{i,t}$$ (2)

In the second stage, we regress *Y*_*i*,*t*_ on ${\hat{x}}_{i,t-1}$(see Eq 3).

$$Y_{i,t}=\beta_{0}+\beta_{1}{\hat{x}}_{i,t-1}{+u\;}_{i,t}$$ (3)

If the variations in the endogenous variables are not explained sufficiently well by the IVs, there could be large standard errors in the IV estimates. These IVs would be described as weak instruments, and the coefficients will be biased like the Ordinary Least Squares [48].

### Distribution dynamics approach

The regression-based approach has been criticized as having significant shortcomings [12]. It does not capture the income dynamics and can mask the presence of convergence clubs and the polarization of regions into rich and poor. An alternative framework has been proposed, that is, the transition probability matrix (TPM) approach based on the Markov chain process. The advantage of this method is that it formulates a law of movement for the entire distribution of incomes allowing us to model the existence of convergence clubs. The TPM exhibits the transition from one state of relative income to the same or another state of relative income over time. It, therefore, measures the probability with which the income level in a country or region rises, falls or remains unchanged between the two periods [49].

A first-order Markov chain is a mathematical model for stochastic systems whose states, discrete or continuous, are governed by a transition probability as follows:

$$\emptyset_{t}=M.\emptyset_{t-1}$$ (4)

where “M” maps the transition between the income distributions for two periods “t” and “t-1” (S4 Text). The probability density distribution “ϕ_t_” is expected to evolve according to the Markov process. We use a first-order Markov process as the density distribution “ϕ” for the period “t” is made dependent on the “ϕ” of the immediately preceding period “t-1” for a finite number of states.

There are three possible trajectories for an economy over a given period of time:

1. It may move ahead (poor catch up with the richer states)—convergence,
2. it may stay where it was, or
3. it may fall behind—divergence.

One advantage of this method is that it is a nonparametric approach and does not rely on the normality assumption of regression-based approaches. However, this method has the limitation that it is unable to examine the factors that cause growth.

In this paper, we use both these approaches to understand the determinants of growth and convergence. We offer a methodological improvement by using the IV approach to control for endogeneity in the regression-based approach. We also use the ‘distribution dynamics’ methodology to confirm that our findings of divergence from the regression analysis are robust to methodological diversity.

India has a three-tiered structure of government—the center, states, and local governments. Over the period of our study, two political parties were dominant at the national level—the Indian National Congress and the Bharatiya Janata Party. Although regional parties have played a significant role at the state-level from 1950, the Indian National Congress enjoyed a parliamentary majority barring two periods [50]. States in India have their own elected governments, whereas UTs are governed by an administrator appointed by the President. There could be considerable center-state conflict when the ruling party in a state is not an ally of the national ruling party.

A large number of studies have tested a heterogeneous set of political variables such as measures of democracy, government stability, political violence, political volatility, and subjective measures of politics in growth regressions [51–54]. In India with its federal structure, some have suggested that discretionary transfers (development assistance transfers) from the center to a state are larger and accelerated if there was an alliance between the party in power at the state and the center [55]. This amount was independent of the development expenditures undertaken by the state with its own resources. We use the number of years that the state party is an ally of the party at the center as an explanatory variable as it may represent the many discretionary benefits that a “friendly” state government may be privileged to. We checked if the political variable could be an instrument and found that the number of years the center and the state parties are allies is independent of other variables in the growth model as there is no significant correlation between them (S2 Table). We also found that the per capita grants under the centrally sponsored schemes were higher as the years of political alliance increased. We use an additional variable from the political domain—political stability index. Stability in government could be growth enhancing in multiple ways including increased investment, predictability of policies, etc. [51,53]. We use the number of years a party has been in power in a five-year period to construct an index of political stability. If the party has covered the entire five-year term, the index takes the value 1 and decreases proportionately depending on the number of years in power.

We are aware that different social and political movements shape the political process. Indian states have experienced different types of social reform movements which have shaped the sociopolitical institutions of each state. The functioning of decision-making bodies has transformed in states where effective mobilization by caste groups has taken place. The northeastern states are all ST dominated, with each state having its unique ethnic identity. Social reforms and political protests against upper-caste domination, particularly in Bihar, Kerala, Maharashtra, Tamil Nadu, and Uttar Pradesh, have transformed the social and economic status as well as educational outcomes for the marginalized groups, the backward classes, and women. They have influenced access to primary education, occupation, landholdings, asset, and livestock for certain states in India [56–58]. India’s Affirmative Action program has conventionally been caste-based with quotas in government jobs and higher education based on caste proportional to their share in the population (though state quotas vary and may not be according to the percentage of population) [59,60]. We have used the proportion of each marginalized caste group in different states as an explanatory variable and instrument. We tested the correlation between the marginalized groups and other variables in the growth model and found no significant correlation between them (S2 Table).

### Data

We use the per capita Net State Domestic Product (PCNSDP, henceforth PCI) series from the Economic and Political Weekly Research Foundation (EPWRF) database. State-level income data for all the states and UTs are available from 1981 which provide us with 30 years of data for 28 states and UTs. In order to control for the price variability over time, we use a price deflator to derive real PCI at the state level. The deflator was obtained by dividing India’s Net Domestic Product (NDP) at current prices in each year by NDP at constant prices (base 2004–05 prices). The 30-year period (from 1981 to 2010) was split into six, five-year subperiods, that is, 1981–85, 1986–90, 1991–95, 1996–00, 2001–05, and 2006–10. This provided us with a panel data set for 28 states (6x28 observations). In each subperiod the initial PCI is its value at the beginning of each five-year period. For example, the growth equation for 1981–1985 would use the PCI of 1981 as an explanatory variable. The gap between the initial value and its terminal value (1985) in this case represents the growth in each subperiod and is divided by 5 to get annual average. The natural log value of PCI is used for the analysis and referred as lnPCI in the rest of this paper.

In the year 2000, there was a reorganization of states in India. Chhattisgarh, Jharkhand, and Uttaranchal (now called Uttarakhand) were carved out of the existing states of Madhya Pradesh, Bihar, and Uttar Pradesh, respectively, to create three new states. We combined these newer states with their parent states to allow for time series compatibility.

Data on demographic variables and social stratification have been taken from the Census of India. The four major social groups for which data are available are the SCs, STs, OBCs, and the Others or General (which includes everyone else). Up to the mid-1990s, the Census of India provided data of three broad social categories: SCs, STs, and the Others (which meant everyone else). After the mid-1990s the subcategory ‘Others’ was further divided into ‘OBCs’ and the remaining were labeled ‘Others’. OBCs are the “socially and educationally” backward classes according to Article 15 of the Indian Constitution. OBCs constitute a heterogeneous collection of Hindu lower castes, some non-Hindu communities and some tribes which are not included in the ST list [59]. In this paper, however, we have not considered the OBC category due to the following reasons: (a) The Census of India does not provide data on OBCs from the period 1981 (the initial period under study); and (b) the identification of the OBCs is an ongoing process, with the number fluctuating over time. The population proportion categorized as SC and ST has been comparatively fairly stable for the entire period of our study. We have accordingly used the data on caste (SC, ST, and others) for the years 1981–2010 from the Census of India (S5 Text and S3 Table).

In the Indian administrative classification of states, there are two categories: general and special. The special category states were characterized by hilly terrain and high density of tribal population, among other criteria. The states of the northeast belong to this category and, therefore, we have created a categorical variable Ne_ST to distinguish these states from the others. We have extrapolated the mid-decade value of the demographic variables from the decadal values.

The data on development expenditure are taken from the EPWRF (“Finances of the state governments” module) for the respective years. The political variable data are taken from the online site http://www.elections.in which is a repository for election results in India.

We now present the results of regression models and the transition probability matrix in the next section.

## Results

Our summary statistics suggest that the average growth rate during the period of our study is 0.05 and the average lnPCI is 9.65 (Table 1). Similarly, the per capita development expenditure (lag_pcdevexp) on an average is Rs 1,175. The SC and ST average is reported as 12.1 percent and 19.5 percent, respectively. The average number of years of political alliance (pol) is three years, with the average of working age (wkage) group being 56.6 years, and the political stability index (stable_index) is 0.9.

**Table 1:** Descriptive statistics of variables used in the analysis.

Variables	Obs	Mean	Std dev	Min	Max
GR	160	0.05	0.03	-0.02	0.13
lnPCI (Rs)	160	9.7	0.51	8.6	11.22
ST (northeast states) (%)	160	14.8	28.9	0	94.8
ST (%)	160	19.5	27.4	0	94.8
SC (%)	160	12.1	8.316	0	30.4
Political alliance (years)	160	3	1.9	0	5
Square of political alliance	160	12.5	10.1	0	25
Political stability index	160	0.89	0.2	0.6	1
Working age group (years)	160	56.6	5.4	29.6	67.9
Per capita development expenditure (in Rs)	132	1174.8	1118.4	0	5178

Data from EPWRF, Census, https://www.elections.in/, and authors’ calculations.

We begin by presenting the results of our first model in Eq 1 where growth is explained by initial income alone. As discussed earlier, panel data models overcome problems that cross-section models and the pooled Ordinary Least Squares models are unable to tackle. We use the Hausman test to decide on the appropriateness of the random effects panel data model. The results confirm that the fixed effect model is appropriate (chi-square 4.97, p-value 0.02) for convergence models using our data as has been anticipated in the literature [22]. The results suggest that initial income is strongly predicting growth rates and the positive coefficient of initial income suggests divergence of incomes which has been anticipated by the received literature (Table 2: column 2—Model 1).

**Table 2:** Growth model estimates of panel and instrumental variable approach (1981–2010).

Independent variable		Dependent variable—growth rate		
	Fixed effect	Fixed effect	First stage (dependent variable—lnPCI)	Second stage (dependent variable—growth rate)
	(Model 1)	(Model 2)	(Model 3: IV regression (TSLS))	
1	2	3	4	5
lnPCI	0.03*** (3.7)	-0.023 (-1.42)		0.03*** (3.03)
SC ($)		-0.0004 (-0.12)	0.05* (1.89)	
ST ($)		-0.0008 (-1.20)	-0.001 (-0.22)	
Ne_ST ($)		0.007** (2.54)	0.08** (2.54)	
lag_pcdevexp ($)		0.00001** (1.99)	0.0002*** (7.64)	
pol ($)		0.0002 (0.05)	-0.09** (-2.09)	
sq_pol ($)		-0.0002 (-0.20)	0.01* (1.65)	
stable_index ($)		-0.001 (0.08)	0.35** (2.48)	
wkage ($)		0.002 (1.05)	0.04** (2.44)	
Constant	-0.24 (-3.1)	0.04 (0.27)		
Obs/no of states	168/28	132/28	132/28	132/28
R-squared (overall)	0.06	0.005	0.74	-0.001

t-values in parentheses:

*** p<0.01,

** p<0.05,

* p<0.1.

^$^—In model 3, these variables are used as instruments.

Data from EPWRF, Census, and authors’ calculations.

However, this model fails to capture the institutional complexities of a country like India. We now expand this model to include additional explanatory variables like the initial proportion of marginalized groups (SC, ST, and Ne_ST), number of years of political alliance (pol and sq_pol), working age group (wkage), political stability index (stable_index) and per capita development expenditure (lag_pcdevexp) in each state (Eq 5). We use the squared term of the political variable to control for any nonlinearity in the relationship.

$${GR}_{i,t}=\beta_{0}+{{\beta_{1}\;ln(PCI)}_{i,t-1}+}_{}{\beta_{2}SC\;}_{i,t-1}+\beta_{3}{ST}_{i,t-1}+\beta_{4}{Ne\_ST}_{i,t-1}+\beta_{5}{lag\_pcdevexp}_{\;i,t-1}+{\beta_{6}pol\;}_{i,t-1}+{\beta_{7}sq\_pol\;}_{i,t-1}+\beta_{8}{wkage}_{i,t-1}{+\beta_{9}{stable\_index}_{i,t-1}+u}_{i,t-1}$$ (5)

When we use an expanded growth equation, the impact of initial income is negative but not significant. All the new variables introduced in the model (that is, SC, ST, and sq_pol) are insignificant and negative, while the variables (stable_index, pol, wkage) are insignificant and positive. On the other hand, Ne_ST and lag_pcdevexp are positive and significant (Table 2: column 3—Model 2).

It is possible, as discussed earlier, that the coefficients could be biased in such a model. We used the chi-square test to check for endogeneity. Our results confirm that the null hypothesis of exogeneity cannot be accepted (chi-square value 7.004 with a p-value of 0.008; S4 Table). In order to overcome the problem of endogeneity we used the TSLS regression method. The proportion of marginalized groups (SC, ST, and Ne_ST), pol, sq_pol, stable_index, wkage, and lag_pcdevexp are used as instruments to predict initial income (lnPCI) in the first stage equation (refer earlier Eq 2 and see 6 below):

$${ln\hat{\left(PCI\right)}}_{i,t-1}=\alpha_{0}+\alpha_{1}{SC\;}_{i,t-1}+\alpha_{2}{ST}_{i,t-1}+\alpha_{3}{N{e\_ST}_{}}_{i,t-1}+{\alpha_{\;4}{lag\_pcdevexp}_{i,t-1}}_{}+{\alpha_{5\;}{pol}_{}}_{i,t-1}+{\alpha_{6}sq\_pol}_{\;i,t-1}+\alpha_{7}{wkage}_{i,t-1}++\alpha_{8}{stable\_index}_{i,t-1}$$ (6)

The results of the first stage regression show a positive and significant relationship between the lnPCI and the instruments (Table 2: column 4—Model 3) except the pol (negative and significant) and ST (negative and insignificant) confirming that there is a nonlinear relationship between initial lnPCI and pol. The political alliance between the center and state has a negative impact on initial income but there are thresholds and longer alliances generate higher initial incomes. We find a U-shaped relationship between initial income and length of political alliance. The positive sign for coefficients of SC and Ne_ST variables implies that a higher percentage of these groups had a positive effect on initial lnPCI. The coefficient of lag_pcdevexp is positive and significant indicating that higher development expenditures have a positive impact on the initial lnPCI.

The model is over-identified by five degrees of freedom, as there is one endogenous regressor and eight excluded instruments. We use the Stock and Yogo test for weak instruments to estimate the F-statistic form of the Cragg-Donald (CD) statistic (S6 Text) [48]. The null hypothesis that the estimator is weakly identified is rejected as the calculated F-value exceeds the maximal value in the confidence interval of CD statistic. This suggests that the instruments in our model are not weakly identified. The J-statistic (with a p-value greater than 0.1) indicates that the null hypothesis of over-identifying restrictions is not rejected.

In the second stage of the TSLS regression (Table 2: column 4—Model 3), the predicted value of lnPCI is used in the original structural equation (refer earlier equation 3 and see 7 below):

$${GR}_{it}=\beta_{0}+\beta_{1}{\hat{lnPCI}}_{i,t-1}+u_{it}$$ (7)

We report the Generalized Method of Moments (IV-GMM) estimates as our model has an over-identified equation and the IV-GMM estimates with robust standard errors will be more efficient than the TSLS or the Limited Information Maximum Likelihood (IV-LIML) estimates [61]. The results of the IV-GMM model reconfirm that initial income has a positive effect on growth implying divergence among the states in India (Table 2: column 5—Model 3).

### Analysis

The popular perception that the presence of large proportions of lower caste is a hindrance to growth is contradicted by our regression results. The question that can be raised is: How reliable or robust are these econometric results to suggest that the presence of lower castes is not a hindrance to growth? After all, the regression-based approaches have their limitations and these results do not give an insight into distributional dynamics at the state level. We, therefore, cross-validate our regression results by using the distribution dynamics approach.

We present a matrix for the two marginalized groups (SC and ST) for the period under study (Table 3). The purpose is to examine which of the states have exhibited mobility in their relative PCI status. In this part of the analysis, we have not separated out the northeastern states from the other states. The national average of the caste categories are 16.6 percent for SCs and 8.6 percent for STs for the Census year 2011 as stated earlier. We classify all states into two categories: those that have more than the national average of marginalized groups (dominant); and those that have less than the national average. The states are grouped according to their relative PCI. The matrices have four rows and four columns. The first group (row 1 and column 1) represents all states who have a PCI that is less than 75 percent of the national average. The second group represents all states who have a PCI between 75 percent and the national average. The third group has all states with PCI above the national average but less twice the national average. Finally, the fourth group has states that has PCI more than double the national average.

**Table 3:** Relative per capita income transition dynamics, 1981–2010.

		2010				Number of states
		States with ending level <0.75	States with ending level = or >0.75 but <1 or = 1	States with ending level >1 but <2 or = 2	States with ending level >2	
1981	States with starting level <0.75	0.333 Bihar, MadhyaPradesh, Odisha	0.556 Meghalaya, Mizoram, Tripura, Rajasthan, Tripura, UttarPradesh	0.111 Kerala		9
	States with starting level = or >0.75 but <1 or = 1	0.272 Assam, Jammu&Kashmir, Manipur	0.090 WestBengal	0.636 AndhraPradesh, ArunachalPradesh, HimachalPradesh, Karnataka, Nagaland, Sikkim, TamilNadu		11
	States with starting level >1 but <2 or = 2			0.857 Gujarat, Haryana, Maharashtra, Punjab, Puducherry, Andaman and Nicobar Islands	0.143 Goa Goa	7
	States with starting level >2				1 Delhi	1

Authors’ calculation based on EPWRF data.

States with SC >16.6 percent marked in blue color font, states with ST >8.6 percent marked in orange color font, and states with both SC>16.6 and ST>18.6 marked in red color font and italicized.

In the matrix, the states above the diagonal have improved their relative PCI from its initial relative position. States that are located below the diagonal have dropped in their relative PCI as compared to the initial period. And those located on the diagonal have not changed from their initial relative position.

In Table 3 we present the transition matrix for the period 1981 to 2010. The first column contains the number of states in each income group in 1981 and the first row contains the number of states in each income group in 2010.

The states with a large SC population (more than 16.6 percent in 2011) have been categorized as dominant (marked in blue for ease of identification, Table 3). Among the states that have dominant SC population, we find that Tripura, Uttar Pradesh, and Rajasthan have moved above the diagonal, but they are still at the lower end of the income levels. Himachal Pradesh has shown a remarkable improvement, besides the southern states of Karnataka and Tamil Nadu. The movement of the southern states above the diagonal is in conformity with the literature which argues that social reforms in the southern states have brought about significant social development despite the presence of large proportion of lower classes [9].

In order to check for dynamics in the ST-dominated states, we treat those which have more than the national average of 8.6 percent of ST population in the year 2011 as dominant (marked in orange, and the states with both dominant SC and ST are marked in red and italicized for ease of identification). The northeastern states have a high proportion of ST population and most of these states, except Assam and Manipur, have moved above the diagonal showing an improvement in their relative per capita levels.

The results of the distribution dynamics approach confirm that some of the SC- and ST-dominated states have improved their relative PCI. This is more pronounced in the southern states. This validates our claim and confirms our findings from the regression-based approach that the presence of large proportions of SC or ST populations do not hinder growth.

## Discussion

The received literature on growth empirics in India has not addressed the issue of endogeneity in regression analysis [47,62]. We find the presence of endogeneity in our expanded growth model. In order to control for endogeneity, we have used the IV approach which is a methodological improvement over the earlier studies. We also find that the regression-based approaches require the fulfilment of the normality assumption [12,63]. The distribution approach overcomes this problem. We find that political factors do play a role in determining growth. First, an alliance between the party at the state and the center is conducive to higher initial income and the relationship is U-shaped. Second, political stability (number of years a party is in power) also has a positive impact on initial income. In terms of policy variables, we find that development expenditure has a positive and significant impact on initial income. The proportion of working age group in the total population also has a positive impact on initial income. We find no evidence that the presence of a large proportion of marginalized groups has adversely affected incomes at the state level.

It has been anticipated in the literature that political alliances yield dividends to states in a federal set-up [64,65]. We find confirmation of this in our results. In a federal structure where devolution of tax revenues is a negotiated outcome between the center and the states, it is rational for political alliances to yield dividends and the electorate would anticipate that.

There is a large political economy literature which suggests that political stability brings positive growth synergies [66,67]. We find confirmation of this in the Indian context. Multiple reasons are cited for this and we have not explored this. It probably provides a stable policy environment where long term investment could be planned by producers [67–69].

Very often, states tend to cut development expenditures in India as a first response to fiscal stringency [70]. Our findings suggest that such policies could be counter-productive to growth outcomes. India is currently expecting to gain a demographic dividend given that it has a large proportion of its population in the working age group [71,72]. Our results suggest that growth will be positively impacted by the proportion of the working age population and this would be growth enhancing. We also find that the presence of large marginalized groups in a state does not seem to weaken the growth process. The reasons for this are not explored in this paper and would be an area of future research interest.

Empirical studies on growth in India have a common limitation with regard to availability of detailed data at the subnational level over long periods. The state is the smallest geographical unit for which income data are publicly available from official sources for the period under study and the country as a whole. Socioeconomic data, on the other hand, are available from even subdistrict levels but only at decadal intervals from the Census. Disaggregated data on human capital formation and physical capital at the subnational level are also not available in a systematic manner. These data limitations impose restrictions on detailed analysis of regional growth.

Our findings, we believe, have important implications for the growth literature and public policy in the context of development expenditure and affirmative action in India. Policies that ensure stability of governments, higher development expenditures and human capital would ensure better growth prospects in India.

[The authors would like to acknowledge helpful suggestions on an earlier version of this paper from William Joe, the academic editor of the journal, and two anonymous reviewers of the journal who have helped improve the paper significantly. We are grateful to Anjali Dar Sengupta for editorial support.]

## Supporting information

S1 Text Validity of instruments. (DOCX)

S2 Text Normality tests. (DOCX)

S3 Text Two-stage least square method. (DOCX)

S4 Text Markov transition matrices. (DOCX)

S5 Text Notes on social stratification data. (DOCX)

S6 Text Identification issues. (DOCX)

S1 Table Normality tests. (DOCX)

S2 Table Correlation matrix. (DOCX)

S3 Table Test for variation in SC, ST. (DOCX)

S4 Table Endogeneity test of endogenous regressors. (DOCX)
