# Supplementary material for: “Understanding growth convergence in India (1981–2010): Looking beyond the usual suspects”
Source: PLoS One. 2020 Jun 2;15(6):e0233549. doi: 10.1371/journal.pone.0233549 (PMC7266299; doi:10.1371/journal.pone.0233549)
Supplement: S1 Text — (DOCX) [file pone.0233549.s001.docx]

### S1 Text. Validity of instruments

The instrument Z_i_ is said to be valid if it satisfies three conditions [37]:

Z*_i_* should be strongly correlated with the endogenous variable (x_i_).

(A1) Instrument relevance: corr ($Z_{i}x_{i}$) $\neq0$

Z*_i_*, should be uncorrelated with the error term

(A2) Instrument exogeneity: corr ($Z_{i}u_{i}$) = 0

and

*Z_i_* does not directly impact the dependent variable *Y_i_.*

(A3) corr ($Y_{i}{, Z}_{i} / x_{i}$) = 0

In order to ensure appropriate choice of instruments, we examined the correlation between the variables used in the regression. We present below the correlation matrix between the major variables in the regression at 1 percent level of significance (only relevant values). Evidently, Growth (GR) is only correlated to initial income (lnPCI). The endogenous variable lnPCI is strongly correlated to wkage and lag_pcdevexp (lagged by one period). These fulfil the requirement for being considered as potential instruments. Social categories (SC and ST) and political alliance (pol and sq_pol) are not correlated to either GR or lnPCI.
