# Supplementary material for: “Understanding growth convergence in India (1981–2010): Looking beyond the usual suspects”
Source: PLoS One. 2020 Jun 2;15(6):e0233549. doi: 10.1371/journal.pone.0233549 (PMC7266299; doi:10.1371/journal.pone.0233549)
Supplement: S2 Text — (DOCX) [file pone.0233549.s002.docx]

### S3 Text. Two-Stage Least Square method

The Two-Stage Least Square method first regresses x_i_ on Z_i_, to obtain the predicted values for *x_i_*, and then regresses *Y_i_* on predicted *x_i_.* The coefficient *β_i_* on predicted *x_i_* is expected to be consistent [62].
