# Supplementary material for: “Understanding growth convergence in India (1981–2010): Looking beyond the usual suspects”
Source: PLoS One. 2020 Jun 2;15(6):e0233549. doi: 10.1371/journal.pone.0233549 (PMC7266299; doi:10.1371/journal.pone.0233549)
Supplement: S4 Text — (DOCX) [file pone.0233549.s004.docx]

### S4 Text. Markov transition matrices

For the Markov transition matrices, we assume that the probability of variable s_t_ taking a particular value depends only on its past value *s_t-1_* according to the first-order Markov chain

(A4) *P{s _t_ = j │ s _t-1_ = i }=* $P_{ij}$

where P*_ij_* indicates the probability that state “I” will be followed by state “j”. Since the sum of the probabilities of all the events is equal to 1, it implies that

(A5) *P_i1_+P_i2_+....+P_in_=1*

The transition matrix constructed is as follows

(A6) *P=* $\left[ \begin{matrix} \begin{matrix} P₁₁ & P₁₂ & \cdots P_{1n} \\ P₂₁ & P₂₂ & \cdots P_{2n} \\ \ldots. & \ldots. & \cdots\cdots\\ \ldots. & \ldots. & \cdots\cdots\\ P_{n1} & P_{n2} & \cdots P_{nn} \end{matrix} \end{matrix} \right]$

where row i and column j indicate the probability that state i will be followed by state j.

By iterating the equation

(A7) $\emptyset_{t}=M . \emptyset_{t-1}$

and leaving out the error term, we get

(A8) $\emptyset_{t}=M^{s} . \emptyset_{t-s}$

When we use the Markov chains to model the evolution of relative incomes’ distribution, we are considering each state of the transition probability matrix as the category of relative income. We thus identify the position of the economy in the income distribution at the starting period. This is done by dividing the income distribution into “income states” indicating a range of income levels. We then observe how many of the economies which are in an income state—say, between 0.75 and 1 in the initial period—remain in that very state, or shift elsewhere in the next time period. If they end up in another income state, there is said to be mobility; if they remain in the same state, it represents persistence. The probabilities obtained give us the percentages of economies or regions which, given a starting state, have moved on to a different state.
