# Supplementary material for: “Understanding growth convergence in India (1981–2010): Looking beyond the usual suspects”
Source: PLoS One. 2020 Jun 2;15(6):e0233549. doi: 10.1371/journal.pone.0233549 (PMC7266299; doi:10.1371/journal.pone.0233549)
Supplement: S5 Text — (DOCX) [file pone.0233549.s005.docx]

### S5 Text. Notes on social stratification data

We have used caste data from the Census. The other potential source of demographic data is the National Sample Survey Office. Its quinquennial surveys have a higher frequency (every five years) than the Census (every decade). However, the National Sample Survey Office quinquennial survey data on caste are available from 1993–94 onwards, namely the 50^th^ (1993–94), 55^th^ (1999–2000), 61^st^ (2004–05), and 66^th^ (2009–10) rounds. We have, however, not used this source as it is available only for a shorter period which did not lend itself to trend analysis.
