# Supplementary material for: “Understanding growth convergence in India (1981–2010): Looking beyond the usual suspects”
Source: PLoS One. 2020 Jun 2;15(6):e0233549. doi: 10.1371/journal.pone.0233549 (PMC7266299; doi:10.1371/journal.pone.0233549)
Supplement: S6 Text — (DOCX) [file pone.0233549.s006.docx]

### S6 Text. Identification issues

In the Instrumental Variable (IV) approach, the relation between the number of instruments (Z) and the number of endogenous regressors (*k*) decides whether the coefficients are identified or not [62]. We require at least one instrument for one endogenous variable. There could be three possibilities wherein the coefficients could be:

1. Just or Exactly Identified: *(Z=k)* if there is one instrument for each of the endogenous variables. In this case the estimator would be unbiased.
2. Under-identified: *(Z>k)* if there are few instruments to estimate the endogenous regressors.
3. Over-identified: *(Z<k)* if there are more instruments than the endogenous variables. This is a desirable option. However, it is important to test if the instruments are valid. Thus, in the IV approach, a valid instrument would isolate the part of the endogenous variable that is not related with the error term and this part is used to estimate the impact of change in the endogenous variable on the dependent variable.

To test for the presence of weak instruments, one approach that has been advanced by Stock and Yogo [46] is the F-statistic form of the Cragg and Donald (CD) (1993) statistic [63]. In our result the weak identification test value (CD Wald F-statistic) is 27.4, while the Hansen J statistic (over-identification test of all instruments) is 3.4 with the chi-sq (3) P-val = 0.3254. This allows us to reject the null hypothesis that the instrument are under-identified.
