# Supplementary material for: “Understanding growth convergence in India (1981–2010): Looking beyond the usual suspects”
Source: PLoS One. 2020 Jun 2;15(6):e0233549. doi: 10.1371/journal.pone.0233549 (PMC7266299; doi:10.1371/journal.pone.0233549)
Supplement: S1 Table — (DOCX) [file pone.0233549.s007.docx]

### S1 Table: Normality tests

Shapiro-Wilk W test for normal data

-------------+------------------------------------------------------

Variable | Obs W(or W’) V(or V’) z Prob>z

-------------+------------------------------------------------------

swilk r | 140 0.98 2.17 1.75 0.039

sfrancia r | 140 0.97 2.89 2.15 0.015

-------------+------------------------------------------------------
