# Supplementary material for: “Understanding growth convergence in India (1981–2010): Looking beyond the usual suspects”
Source: PLoS One. 2020 Jun 2;15(6):e0233549. doi: 10.1371/journal.pone.0233549 (PMC7266299; doi:10.1371/journal.pone.0233549)
Supplement: S2 Table — (DOCX) [file pone.0233549.s008.docx]

**S2 Table: Correlation Matrix**

-------------+----------------------------------------------------------------------- | | GR lnpcnsdp st sc pol sq_pol wkage lag_pcdev stable_

-------------+-----------------------------------------------------------------------

GR | 1.00

lnpcnsdp | 0.23* 1.00

st | 1.00

sc | -0.62* 1.00

pol | 1.00

sq_pol | 0.96* 1.00

wkage | 0.61* 1.00

lag_pcdevexp | 0.46* 0.38* -0.25* 1.00

stable_index | 1.00

-------------+-----------------------------------------------------------------------

Note: Only significant correlation coefficients are reported.
