# Supplementary material for: “Understanding growth convergence in India (1981–2010): Looking beyond the usual suspects”
Source: PLoS One. 2020 Jun 2;15(6):e0233549. doi: 10.1371/journal.pone.0233549 (PMC7266299; doi:10.1371/journal.pone.0233549)
Supplement: S3 Table — (DOCX) [file pone.0233549.s009.docx]

**S3 Table: Test for Variation in SC, ST**

We tested for variation in SC and ST. The summary tables below indicate a reasonable variation in the two covariates (SC and ST).

-------------+------------------------------------------------------

Variable Obs Mean Std. Dev. Min Max

-------------+------------------------------------------------------

SC 168 11.79 8.4 0 30.4

ST 168 19.48 27.3 0 94.75

-------------+------------------------------------------------------
