# Supplementary material for: “Understanding growth convergence in India (1981–2010): Looking beyond the usual suspects”
Source: PLoS One. 2020 Jun 2;15(6):e0233549. doi: 10.1371/journal.pone.0233549 (PMC7266299; doi:10.1371/journal.pone.0233549)
Supplement: S4 Table — (DOCX) [file pone.0233549.s010.docx]

S4 Table: Endogeneity test of endogenous regressors

| 1 | Endogenous regressor | lnPCI |
| --- | --- | --- |
| 2 | Excluded instruments and regressors tested | SC, ST, Ne_ST, pol, sq_pol stable_index, wkage, lag_pcdevexp |
| 3 | chi-sq(1) | 7.004 |
| 4 | P-value | 0.008 |
